# Retraction Note: Progesterone receptor assembly of a transcriptional complex along with activator protein 1, signal transducer and activator of transcription 3 and ErbB-2 governs breast cancer growth and predicts response to endocrine therapy

**DOI:** 10.1186/s13058-023-01735-z

**Published:** 2023-11-02

**Authors:** María C. Díaz Flaqué, Natalia M. Galigniana, Wendy Béguelin, Rocío Vicario, Cecilia J. Proietti, Rosalía Cordo Russo, Martín A. Rivas, Mercedes Tkach, Pablo Guzmán, Juan C. Roa, Esteban Maronna, Viviana Pineda, Sergio Muñoz, María Florencia Mercogliano, Eduardo H. Charreau, Patricio Yankilevich, Roxana Schillaci, Patricia V. Elizalde

**Affiliations:** 1 Instituto de Biología y Medicina Experimental (IBYME), CONICET, Obligado 2490, 1428 Buenos Aires, Argentina; 2 Universidad de La Frontera, Temuco, Chile; 3 Sanatorio Mater Dei, Buenos Aires, Argentina; 4 y, Instituto de Investigación en Biomedicina de Buenos Aires (IBioBA), CONICET - Partner Institute of the Max Planck Societ, Buenos Aires, Argentina

**Retraction Note: Breast Cancer Research 2013, 15**:R118 10.1186/bcr3587

The Editor-in-Chief has retracted this article. After publication, concerns were raised regarding the western blot images presented in the figures. Specifically:

- In Fig. 6g, the two pErbB2 blots appear highly similar, including the background features, with a couple of bands showing differences. In the pErbB2 (Tyr927), lanes 6 and 10 appear highly similar, and there appears to be a splice mark to the left of lane 10.
- In Fig. 6g pp44/pp42 MAPK blot, lanes 3 and 4 appear highly similar to lanes 9 and 10, and there appears to be a splice mark to the left of lane 9.
- In Fig. 6g p42/p44 MAPK blot, lanes 1 and 5 appear highly similar.
- In Fig. 8a, there appears to be a splice mark in the p–c-Jun blot between lanes 2 and 3. There are no splices in the c-Jun blot.
- In Fig. 8f and g, three of the four p42 and p44 MAPK western blot bands appear highly similar, although the figures represent different cells.

The Editor-in-Chief therefore no longer has confidence in the presented data.

María C Díaz Flaqué, Natalia M Galigniana, Patricio Yankilevich and Patricia V Elizalde do not agree to this retraction. None of the other authors have responded to any correspondence from the editor or publisher about this retraction.
